# Ambiguity, Ambivalence, and Apprehensions of Taking HIV-1 Pre-Exposure Prophylaxis among Male Couples in San Francisco: A Mixed Methods Study

**DOI:** 10.1371/journal.pone.0050061

**Published:** 2012-11-14

**Authors:** Parya Saberi, Kristi E. Gamarel, Torsten B. Neilands, Megan Comfort, Nicolas Sheon, Lynae A. Darbes, Mallory O. Johnson

**Affiliations:** 1 University of California San Francisco, San Francisco, California, United States of America; 2 Graduate Center at the City University of New York, CUNY, New York, United States of America; 3 RTI International, San Francisco, California, United States of America; The University of New South Wales, Australia

## Abstract

**Objective:**

We conducted a mixed-methods study to examine serodiscordant and seroconcordant (HIV-positive/HIV-positive) male couples' PrEP awareness, concerns regarding health care providers offering PrEP to the community, and correlates of PrEP uptake by the HIV-negative member of the couple.

**Design:**

Qualitative sub-study included one-on-one interviews to gain a deeper understanding of participants' awareness of and experiences with PrEP and concerns regarding health care providers offering PrEP to men who have sex with men (MSM). Quantitative analyses consisted of a cross-sectional study in which participants were asked about the likelihood of PrEP uptake by the HIV-negative member of the couple and level of agreement with health care providers offering PrEP to anyone requesting it.

**Methods:**

We used multivariable regression to examine associations between PrEP questions and covariates of interest and employed an inductive approach to identify key qualitative themes.

**Results:**

Among 328 men (164 couples), 62% had heard about PrEP, but approximately one-quarter were mistaking it with post-exposure prophylaxis. The majority of participants had low endorsement of PrEP uptake and 40% were uncertain if health care providers should offer PrEP to anyone requesting it. Qualitative interviews with 32 men suggest that this uncertainty likely stems from concerns regarding increased risk compensation. Likelihood of future PrEP uptake by the HIV-negative member of the couple was positively associated with unprotected insertive anal intercourse but negatively correlated with unprotected receptive anal intercourse.

**Conclusions:**

Findings suggest that those at greatest risk may not be receptive of PrEP. Those who engage in moderate risk express more interest in PrEP; however, many voice concerns of increased risk behavior in tandem with PrEP use. Results indicate a need for further education of MSM communities and the need to determine appropriate populations in which PrEP can have the highest impact.

## Introduction

The use of HIV antiretroviral (ARV) medications by HIV uninfected individuals to reduce risk of HIV infection prior to engaging in high risk behavior, also known as HIV-1 pre-exposure prophylaxis (PrEP), has been shown to be effective in four clinical trials to date [1], [2], [3], [4]. The seminal PrEP trial, iPrEx, revealed that tenofovir disoproxil fumarate/emtricitabine (TDF/FTC) can reduce the risk of HIV in men who have sex with men (MSM) and transgender women at high risk for HIV infection by 44% [2]. The Partners PrEP, TDF2, and CAPRISA004 studies have revealed further evidence of the efficacy of PrEP in other populations [1], [3], [4]; however, the FEM-PrEP and two arms of the VOICE trials have been stopped for futility [5], [6], [7].

Despite these promising results, MSM communities continue to have ambivalence and concerns about PrEP. Prior studies have examined the knowledge of and attitudes toward PrEP use [8], [9], [10], [11]; however, due to the novelty of PrEP, many questions regarding the acceptability and adoption of PrEP within the MSM community and correlates of PrEP uptake remain unanswered. Serodiscordant couples represent an important target group for PrEP, thus it is important to understand perceptions and attitudes of both HIV-negative and HIV-positive partners towards PrEP as such understanding will contribute to optimizing uptake when PrEP becomes available.

While PrEP has high potential as a biomedical HIV prevention intervention strategy [12], [13], [14], [15], effective policies and programs to support PrEP will require the input of MSM communities. Moreover, the approval of TDF/FTC as HIV-1 PrEP by the Food and Drug Administration intensifies the need for knowledge about PrEP awareness, use, and potential barriers to its success. To address gaps in knowledge, we conducted a mixed-methods study to examine the awareness of PrEP to prevent HIV infection, concerns regarding offering PrEP to the community, and correlates of future intentions for PrEP uptake by HIV-negative individuals. Our quantitative approach consisted of a cross-sectional study of HIV serodiscordant and seroconcordant (HIV-positive/HIV-positive) male couples to explore awareness of the existence of PrEP and examine the correlates of the participant's or the participant's partner's likelihood of PrEP uptake in the future and the level of agreement with the notion of health care providers offering PrEP to anyone who requests it. Qualitative interviews were conducted with a subsample of serodiscordant and seroconcordant couples to gain a deeper understanding of participants' knowledge of PrEP, personal experiences with PrEP, and perspectives regarding offering PrEP to the community.

## Methods

### Design

This mixed-methods study draws on data from serodiscordant (HIV-positive/HIV-negative) and seroconcordant (HIV-positive/HIV-positive) male couples of the Duo Project, a longitudinal mixed-methods study of how relationship dynamics influence ARV adherence [16], [17].

In the quantitative survey of the Duo Project, we inquired about participants' PrEP knowledge, potential future PrEP uptake by the HIV-negative member of the couple, and level of agreement with the approach of offering PrEP to anyone who requests it. The qualitative interview guide of the Duo Project included a subsection of questions about participants' knowledge of and experiences with PrEP and concerns regarding health care providers offering PrEP to the community.

### Participants

Eligible participants were men who were 18 years or older, provided written informed consent, had been in a primary relationship for a minimum of three months, and at least one of the men had to be HIV-positive and taking ARV medications for at least 30 days. Those who showed signs of severe cognitive impairment or active psychosis were excluded. Couples were recruited from the San Francisco Bay Area using referrals from participants and passive recruitment for which advertisements were posted on community bulletin boards, clinic waiting rooms, and at community-based organizations. Couples who called the study telephone number were screened separately to confirm eligibility and eligible couples were scheduled for an interview.

The qualitative interviews employed a purposeful sampling strategy [18]. Following participants' 12-month assessment in the Duo Project, couples were systematically selected to participate in a qualitative interview based on adherence reports at their 12-month visit. Consistent with the overarching goals of the Duo Project, our qualitative sampling strategy allowed us to compare seroconcordant (HIV-positive/HIV-positive) and serodiscordant couples with different levels of adherence.

The University of California, San Francisco Committee on Human Research granted approval of this research and participants signed an informed consent form prior to initiation of study procedures.

### Data Collection

#### Data collection for quantitative survey

To minimize partial couple's data (i.e., obtaining data from one member of the couple only), we required couples to attend assessment appointments together, but they were separated during the consenting process and data collection to avoid the possibility of shared responses or potential partner coercion. Data were collected with a combination of Computer Assisted Personal Interviewing (CAPI) and Audio Computer Assisted Self Interviewing (ACASI) procedures.

The PrEP questionnaire was included in the Duo Project questionnaire starting April 2011 and assessed participants' awareness of the existence of PrEP; the sources of their information regarding PrEP; and prior use of PrEP by self, partner, or acquaintance. Participants were initially asked if they had heard of PrEP or the iPrEX study and PrEP was defined as “HIV-negative people taking HIV medications to try to reduce their chances of becoming infected with HIV.” Three key PrEP questions, PrEP1, PrEP2, and PrEP3, were asked in quantitative surveys. PrEP1 asked “How likely is it that your partner would use PrEP in the future?” This question was only asked from participants whose partners were HIV-negative and responses ranged from 0: “not at all likely” to 9: “extremely likely”. The HIV-negative individuals' likelihood of taking PrEP in the future was asked by PrEP2: “How likely is it that you would use PrEP in the future?” Responses ranged from 0: “not at all likely” to 9: “extremely likely”. Lastly, PrEP3 was asked of all participants and assessed participants' level of agreement with offering PrEP: “How much do you agree or disagree with the following statement: ‘PrEP should be offered to anyone who wants to take it’.” With responses ranging from 1: “strongly disagree” to 5: “strongly agree”.

Other information collected on participants included demographics (age, race/ethnicity, sexual orientation, education, and income); depression (assessed using the Center for Epidemiologic Studies Depression (CES-D) scale); length of time together as a couple; sexual behavior (report of unprotected insertive/receptive anal intercourse with primary or other partner) in the past three months; marijuana, illicit substances (crack, cocaine, heroin, and street methadone), erectile dysfunction drugs (while high, partying, or drunk), or stimulant use in the past three months; and HIV clinical parameters for participants who were HIV-positive (months since tested HIV-positive, taking ARV therapy, self-reported CD4+ cell count, and undetectability of HIV viral load).

#### Data collection for qualitative interviews

Data were collected through semi-structured interviews conducted separately but simultaneously with each partner from March through September 2011. Interviews consisted of open-ended questions designed to elicit information about PrEP knowledge and acceptability. PrEP was defined as “HIV-negative people taking HIV medications to try to reduce their chances of becoming infected with HIV.” Participants were asked to describe: 1) what they had heard about PrEP, 2) how they had learned about PrEP, 3) personal experiences they may have had using PrEP themselves or with their partner using PrEP, 4) their discussions with their partner about PrEP, and 5) their thoughts on whether PrEP should be offered to the community at large and potential issues that should be considered in offering PrEP to the community.

### Analysis

#### Quantitative analysis

We used descriptive statistics to generate frequencies for categorical variables and means and standard deviations for continuous variables.

Using bivariable linear regression, we examined the association between the three key PrEP questions (i.e., PrEP1-PrEP3) as outcome variables and covariates of interest such as demographics; sexual behavior; marijuana, illicit substances, erectile dysfunction drugs, or stimulant use; CES-D score; length of time together as a couple; and HIV parameters for HIV-positive participants (months since tested HIV-positive and taking ARVs). All covariates with a p-value<0.25 in the bivariable models were placed in three multivariable linear regression models (Models 1–3), corresponding to the three key PrEP questions [19]. Using backward elimination, variables were removed until all remaining variables had a p-value<0.05. Model assumptions were assessed by fitting restricted cubic splines for each continuous covariate and performing a Wald test of spline terms 2 through k to assess for linearity of the relationship of continuous covariates with each outcome [20] and normality was checked by examining histograms of residuals. Interactions were assessed by fitting all possible first-order interactions in multivariable models at alpha = 0.05 as a preliminary step prior to finalizing each model. For Models 1 and 2, the HC3 heteroskedastic consistent estimator was used to minimize model assumption violations. Due to nesting of individuals within dyads in Model 3, we used robust Huber-White standard errors, clustering on the couple ID. For all regression analyses, we report the unstandardized regression coefficient *B* and its associated p-value. A two-sided p-value<0.05 was considered statistically significant for variables included in the final models. All analyses were conducted using Stata, version 11 (StataCorp, College Station, TX).

#### Qualitative analysis

We employed an inductive approach [21] to gain a deeper understanding of participants' awareness of and experiences with PrEP and their concerns regarding health care providers offering PrEP to members of their community. This approach entails “detailed readings of raw data to derive concepts, themes, or a model”[21]. The interview segments in which participants discussed PrEP were transcribed verbatim and summarized and discussed in detail by the first and second authors (PS and KG). Broad themes were then identified individually by the first and second authors, refined through discussion from the fourth author (MC), and entered into a matrix using Microsoft Excel where each column corresponded to a theme and each row represented a case. This method facilitated data analysis and allowed for the identification of patterns in the distribution of themes [22]. The first and second authors independently categorized each interview using this matrix (inter-rater reliability = 0.88) and coding discrepancies were discussed by the two authors until consensus was reached or arbitrated by the fourth author. Due to the nature of the qualitative study as being nested in the Duo Project, achieving data saturation was not an objective of the qualitative analysis. Rather, we focused on the exploratory goal of eliciting information from the Duo Project participants about their knowledge of, experiences with, and thoughts about using PrEP in their own relationships and concerns about health care providers offering PrEP to the community at large.

## Results

### Quantitative results

#### Demographics

The sample comprised 164 couples (69 serodiscordant and 95 seroconcordant), 259 members of which were HIV-positive and 69 HIV-negative (Table 1). Table 2 summarizes data regarding participants' sexual behavior and drug use in the past three months. With regards to HIV transmission risk behavior, approximately 16% of HIV-positive men in serodiscordant relationships reported having unprotected insertive anal intercourse (UIAI) with their HIV-negative primary partner in the past three months and 5% reported UIAI with someone other than their primary partner who was HIV-negative/serostatus-unknown. Among the HIV-negative men, 13% reported unprotected receptive anal intercourse (URAI) with their HIV-positive primary partner and 3% reported URAI with men other than their primary partner who were HIV-positive/serostatus-unknown.

**Table 1:** Sample characteristics.

	HIV+ (n = 259)	HIV− (n = 69)	Total (n = 328)	p-value
Age, mean (SD)	45.9 (9.4)	44.5 (11.2)	45.6 (9.8)	0.30
Race, n (%)				0.26
Black/African American	42 (16.2)	8 (11.6)	50 (15.2)	
White/Caucasian	152 (58.7)	48 (69.6)	200 (61.0)	
Other	65 (25.1)	13 (18.8)	78 (23.8)	
Latino ethnicity, n (%)	45 (17.4)	9 (13.0)	54 (16.5)	0.39
Sexual orientation, n (%)				0.43
Homosexual	189 (73.0)	47 (68.1)	236 (72.0)	
Bisexual/Other/Not sure	70 (27.0)	22 (31.9)	92 (28.1)	
Education, n (%)				0.20
<High school	9 (3.5)	1 (1.5)	10 (3.1)	
High school	63 (24.3)	16 (23.2)	79 (24.1)	
Some college	74 (28.6)	13 (18.8)	87 (26.5)	
College grad. or higher	113 (43.6)	39 (56.5)	152 (46.3)	
Income, n (%)*				0.16
<$10, 000	45 (18.4)	10 (15.2)	55 (17.7)	
$10,000–19,999	72 (29.4)	13 (19.7)	85 (27.3)	
≥$20,000	128 (52.2)	43 (65.2)	171 (55.0)	
CES-D depression score, mean (SD)	14.7 (11.2)	14.1 (11.8)	14.6 (11.3)	0.52
Months as a couple, mean (SD)	79.9 (77.2)	79.9 (87.2)	79.9 (79.2)	0.63
Months since tested HIV-positive, mean (SD)	169.4 (97.8)	-	-	-
On antiretroviral medications, n (%)	245 (94.6)	-	-	-
CD_4_^+^ cell count, mean (SD)^1,2^	564.7 (248.8)	-	-	-
Undetectable HIV viral load, n (%)^2^	29 (11.2)	-	-	-

* The total of some categorical variables will not sum to the total number of participants in each column due to missing data.

CES-D: Center for Epidemiologic Studies Depression scale; SD: standard deviation.

1 n = 255; ^2^ Self-report.

**Table 2:** Sexual and substance use behavior.

	HIV+ (n = 259)	HIV− (n = 69)	Total (n = 328)	p-value
Any UIAI with primary partner in past 3 months, n (%)*	80 (30.9)	13 (18.8)	93 (28.4)	**0.05**
Any URAI with primary partner in past 3 months, n (%)	77 (29.7)	9 (13.0)	86 (26.2)	**0.005**
Any UIAI with men other than primary partner in past 3 months, n (%)	60 (23.2)	15 (21.7)	75 (22.9)	0.80
Number of other men with whom had UIAI in past 3 months, mean (SD)^1^	4.6 (5.6)	2.8 (2.8)	4.2 (5.2)	0.27
Any URAI with men other than primary partner in past 3 months, n (%)	51 (19.7)	4 (5.8)	55 (16.8)	**0.006**
Number of other men with whom had URAI in past 3 months, mean (SD)^2^	4.3 (7.0)	1.8 (1.0)	4.1 (6.7)	0.17
Any UIAI with HIV discordant/unknown men other than primary partner in past 3 months, n (%)	13 (5.0)	11 (15.9)	24 (7.3)	**0.002**
Number of HIV discordant/unknown other men with whom had UIAI in past 3 months, mean (SD)^3^	2.8 (2.6)	3.1 (2.5)	3.0 (2.5)	0.43
Any URAI with HIV discordant/unknown men other than primary partner in past 3 months, n (%)	23 (8.9)	2 (2.9)	25 (7.6)	0.10
Number of HIV discordant/unknown other men with whom had URAI in past 3 months, mean (SD)^4^	2.4 (3.2)	2.5 (0.7)	2.4 (3.0)	0.17
Marijuana use in past 3 months, n (%)	148 (57.1)	39 (56.5)	187 (57.0)	0.93
Illicit substance use in past 3 months, n (%)^5^	30 (11.6)	12 (17.4)	42 (12.8)	0.20
Erectile dysfunction drug use in past 3 months, n (%)^6^	46 (17.8)	5 (7.3)	51 (15.6)	**0.03**
Stimulant use in past 3 months, n (%)	53 (20.5)	12 (17.4)	65 (19.8)	0.57

SD: standard deviation; UIAI: unprotected insertive anal intercourse; URAI: unprotected receptive anal intercourse.

* 16% of HIV-positive men in serodiscordant relationships reported UIAI with their HIV-negative primary partner in the past three months.

1 n = 75; ^2^ n = 55; ^3^ n = 24; ^4^ n = 25.

5 Illicit substances: heroin, street methadone, crack, cocaine.

6 Erectile dysfunction drug use while high, partying, or drunk.

As shown in Table 3, there were no statistically significant differences between HIV-positive and -negative participants' responses to PrEP questions. Over 62% of all participants had heard about PrEP; predominantly through news articles and websites. A total of ten participants reported having ever taken PrEP to prevent HIV. Approximately one-third of respondents had thought of taking PrEP for themselves or that their partner should take PrEP. On a scale of zero to nine, representing “not at all likely” to “extremely likely” to take PrEP in the future, participants indicated a below average endorsement of PrEP uptake for their partners (mean = 3.3) or themselves (mean = 3.9). Lastly, participants most frequently expressed uncertainty about whether PrEP should be offered to anyone who requests it (40%); however, about 48% cumulatively endorsed “agree” or “strongly agree”, suggesting that some participants were open to the idea of PrEP being made more available.

**Table 3:** Response to PrEP questions.

	HIV+ (n = 259)	HIV− (n = 69)	Total (n = 328)	p-value
“Have you ever heard about HIV-people taking HIV medications to try to reduce their chances of becoming HIV infected? You might have heard of this as PrEP.”, n (%)	161 (62.2)	45 (65.2)	206 (62.8)	0.64
“Where did you hear about PrEP?”, n (%) *				0.97
News article or story	70 (43.5)	22 (48.9)	92 (44.7)	
Website	37 (23.0)	10 (22.2)	47 (22.8)	
Research study/study flier	33 (20.5)	8 (17.8)	41 (19.9)	
Sex partner	11 (6.8)	3 (6.7)	14 (6.8)	
Friend or family member	8 (5.0)	2 (4.4)	10 (4.9)	
My doctor	2 (1.2)	0	2 (1.0)	
“Have you ever taken PrEP to try to prevent getting HIV?”, n (%) *	-	8 (17.8)	-	-
“Before you tested HIV+, did you ever take PrEP to try to prevent HIV?”, n (%) *	2 (1.2)	-	-	-
“Do you know anyone who has taken PrEP?”, n (%) *	38 (23.6)	8 (17.8)	46 (22.3)	0.41
“Has your partner ever taken PrEP to try to prevent getting HIV?”, n (%) *	4 (26.7)	-	-	-
“Before he tested HIV+, did your partner ever take PrEP to try to prevent HIV?”, n (%) *	1 (3.6)	0	1 (2.8)	-
“Have you ever thought about you or your partner taking PrEP to try to prevent HIV?”, n (%) *	18 (38.3)	11 (29.7)	29 (34.5)	0.41
“Have you and your partner ever discussed the possibility of you or your partner taking PrEP to try to prevent HIV?”, n (%) *	9 (50)	4 (36.4)	13 (44.8)	0.47
PrEP1: “How likely is it that your partner would use PrEP in the future?”, mean (SD) 1,2	3.3 (3.1)	-	-	-
PrEP2: “How likely is it that you would use PrEP in the future?”, mean (SD) 1,3	-	3.9 (2.7)		
PrEP3: “How much do you agree/disagree with this statement: ‘PrEP should be offered to anyone who wants to take it’”, n (%) *				0.16
Strongly disagree	6 (3.7)	1 (2.2)	7 (3.4)	
Disagree	10 (6.2)	8 (17.8)	18 (8.7)	
Uncertain	64 (39.8)	18 (40.0)	82 (39.8)	
Agree	49 (30.4)	12 (26.7)	61 (29.6)	
Strongly agree	32 (19.9)	6 (13.3)	38 (18.5)	

* The total of some categorical variables will not sum to the total number of participants in each column due to missing data.

SD: Standard deviation.

1 Scale from 0 = “not at all” to 9 = “extremely likely”.

2 n = 52.

3 n = 45.

#### Bivariable linear regression results

Table 4 summarizes p-values of all covariates that were examined in relation to the three key PrEP outcome variables (i.e., PrEP1, PrEP2, and PrEP3). Variables with a p-value<0.25 are bolded and were examined in multivariable regression models.

**Table 4:** Bivariable analysis p-values.

	PrEP1 (n = 51)	PrEP2 (n = 45)	PrEP3 (n = 206)
Age	**0.005**	0.74	**0.02**
Race	0.33	0.64	0.85
White/Caucasian	ref	ref	ref
Black/African American	0.33	0.92	0.73
Other	0.22	0.35	0.69
Latino ethnicity	**0.17**	0.88	0.75
Sexual orientation	-	-	-
Homosexual	ref	ref	ref
Bisexual/Other/Not sure	0.35	0.91	**0.17**
Education	**0.08**	0.74	0.95
<High school	ref	ref	ref
High school	0.29	0.33	0.77
Some college	0.25	0.36	0.75
College grad. or higher	0.11	0.45	0.69
Income	0.58 a	0.40 ^b^	0.42 ^c^
<$10, 000	ref	ref	ref
$10,000–19,999	0.73	0.20	0.20
≥$20,000	0.37	0.55	0.47
CES-D depression score	0.26	0.87	0.55
Months as a couple	0.48	0.74	0.44
Any UIAI with primary partner in past 3 months	**0.13**	**0.01**	**0.11**
Any URAI with primary partner in past 3 months	**0.10**	0.94	0.62
Any UIAI with men other than primary partner in past 3 months	0.44	0.90	**0.05**
Any URAI with men other than primary partner in past 3 months	0.27	0.41	**<0.001**
Any UIAI with HIV discordant/unknown men other than primary partner in past 3 months	0.93	0.80	**0.11**
Any URAI with HIV discordant/unknown men other than primary partner in past 3 months	0.66	**<0.001**	**0.002**
Marijuana use in past 3 months	0.92	**0.20**	0.67
Illicit substance use in past 3 months e	0.67	0.56	**0.14**
Erectile dysfunction drug use in past 3 months f	**0.22**	0.69	**0.009**
Stimulant use in past 3 months	0.61	0.87	0.26
Months since tested HIV-positive	0.29	-	0.94 ^d^
On ARVs	**0.19**	-	0.86 ^d^

a n = 48; ^b^ n = 43; ^c^ n = 199; ^d^ n = 161.

e Illicit substances: heroin, street methadone, crack, cocaine.

f Erectile dysfunction drug use while high, partying, or drunk.

ARV: antiretroviral; CES-D: Center for Epidemiologic Studies Depression scale; Ref: reference variable; UIAI: unprotected insertive anal intercourse; URAI: unprotected receptive anal intercourse.

PrEP1: “How likely is it that your partner would use PrEP in the future?” (0: not at all to 9: extremely): asked of all participants with an HIV-negative partner.

PrEP2: “How likely is it that you would use PrEP in the future?” (0: not at all –9: extremely): asked of all HIV-negative participants.

PrEP3: “How much do you agree or disagree with the following statement: ‘PrEP should be offered to anyone who wants to take it.’” (1: strongly disagree –5: strongly agree): asked of all participants.

#### Multivariable linear regression results

Model 1 examined correlates of HIV-positive men reporting the likelihood of their HIV-negative partner taking PrEP in the future (i.e., PrEP1). In this analysis, the HIV-positive individuals' younger age (*B* = −0.10; 95% confidence interval [CI] = −0.16, −0.03; p = 0.003), lower level of education (*B* for high school graduate = −3.60, p = 0.23; *B* for some college = -5.13, p = 0.09; *B* for college graduate or higher = −6.00, p = 0.04; overall p = 0.03), any URAI with their HIV-negative primary partner (*B* = 3.02; 95% CI = 0.26, 5.77; p = 0.03), and not being on ARVs (*B* = −3.58; 95% CI = −5.61, −1.56; p = 0.001) had statistically significant associations with HIV-positive participants in serodiscordant relationships reporting that their partner has a higher likelihood of taking PrEP in the future.

Model 2 assessed correlates of HIV-negative men's report of their own likelihood of taking PrEP in the future (i.e., PrEP2). In this model, having UIAI with their HIV-positive primary partner (*B* = 2.15; 95% CI = 0.37, 3.93; p = 0.02) and not having URAI with HIV-positive/serostatus-unknown men other than their primary partner (*B* = −3.52; 95% CI = −4.40, −2.63; p<0.001) were significantly associated with HIV-negative men's higher likelihood of taking PrEP in the future.

In the final model (Model 3), examining the level of agreement with making PrEP widely available, younger age (*B* = −0.01; 95% CI = −0.02, −0.0003; p = 0.045) and any URAI with men other than primary partner (*B* = 0.62; 95% CI = 0.33, 0.90; p<0.0001) had statistically significant associations with endorsement of the statement that PrEP should be offered to anyone who requests it. When Model 3 was refitted to each serostatus groups separately, statistically significant results only emerged in the HIV-positive sub-group.

### Qualitative results

#### Demographics

We interviewed 16 couples (six serodiscordant), comprising 32 men which had a mean age of 48.1 years (SD = 9.7), were 25.0% Black and 53.1% White, and 87.5% self-identified as homosexual. Couples had been in a relationship together for a mean duration of 100 months (SD = 68.6), approximately 9% had not received a high school diploma, and 15.6% had an income less than $10,000 per year. A total of 25 individuals (78.1%) were HIV-positive, of which 88% were on ARVs.

#### Themes

Approximately 81% of the participants stated that they had heard about PrEP; however, examination of the narratives revealed that 27% of these individuals were mistaking PrEP for post-exposure prophylaxis (PEP). One participant referred to PrEP as the “oops, morning-after pill.” Four individuals had personal experiences with PrEP, by either participating or knowing someone who was enrolled in a PrEP trial.

Six prominent themes regarding concerns about offering PrEP to the community at large emerged: 1) increased risky behavior or risk compensation (discussed in 28% of interviews), 2) expense and financial coverage of PrEP medications (19%), 3) ARV toxicity and adverse effects (16%), 4) need for more public education (13%), 5) need for more research on PrEP efficacy and behavioral aspects (9%), and 6) drug resistance (9%).

The most frequently stated concern was the likelihood for increased risky behavior. Participants discussed issues related to potential reduction in condom use, decrease in worries related to HIV infection, and complacency in talking to partners about HIV risks.

“Everyone's going to have unsafe sex if they think they can prevent it because nobody wants to feel a condom; it's no fun… Common sense human nature would say ‘no condom, take the PrEP’.” (age 61, White, HIV-positive, seroconcordant relationship).

“It creates this idea, this way of absolving people of taking necessary precautions like using condoms or wanting to know something about their partners.” (age 35, Black, HIV-positive, serodiscordant relationship).

“I'm terrified that people, instead of taking this drug because they're taking risk would feel more comfortable taking risks because they have the drug. I'm worried about whether it would increase or decrease the instances of HIV infection.” (age 49, White, HIV-negative, serodiscordant relationship).

“It will be this open door that people can have unsafe sex… people think that it [HIV]'s not a disease that kills people anymore.” (age 38, White, HIV-positive, seroconcordant relationship).

Among those who brought up financial issues of PrEP, most worried about the entity that would be required to pay for the medications when they became available.

“If you're a really wealthy guy… who works for an investment bank, you'll be fine; but from a practical standpoint… it's not economically feasible or practical; especially given that funding is getting cut.” (age 47, White, HIV-positive, serodiscordant relationship).

Those who mentioned issues surrounding ARV adverse effects were all HIV-positive and taking ARVs. These interviews reflected a perception that the potential toxicities associated with ARVs outweighed the possible benefits of PrEP.

“[PrEP is] not a great idea because these drugs are not super easy on the body, so from a physical standpoint I think anybody would want to *not* take medications versus take medications… [ARVs] affect liver and affect the kidneys and are full of harsh chemicals and are expensive for people and insurance companies… if you have the option of *not* taking medications versus take medications, it seems odd to me to want to take medications.” (age 35, Black, HIV-positive, serodiscordant relationship).

“I would be sort of against it… because you can be safe with condoms and do you really want to be putting drugs in your body unless you really absolutely need it? There are still questions about the long run effects of HIV meds… I think about what can potentially happen to me down the line after all these years of taking these meds but I have to take them, I have no choice.” (age 45, Latino, HIV-positive, serodiscordant relationship).

The need for more public education was brought up numerous times. This education included basic introduction of the existence and uses of PrEP, as well as the need for continued safer sex counseling.

“The only thing that I'd want to make sure of is the education behind sexual practices because another hot thing out there… [is] where these guys now think that people can get on meds and live a long time, they go out and have a lot of unprotected sex which is really sad but no one really knows what it's like until you're positive… PrEP is a good thing and it is like preventative medicine but there has to be a message of ‘the best practice is still safe sex’.” (age 38, White, HIV-positive, seroconcordant relationship).

Several participants discussed the need for more research to establish whether PrEP is, in fact, effective in HIV prevention. One participant noted the importance of behavioral research to examine the potential for increased risk disinhibition.

“I think that it needs to be studied in terms of psychological as well as medical effects to see whether it increases incidence of unprotected anal intercourse.” (age 49, White, HIV-negative, serodiscordant relationship).

A few HIV-positive participants noted concerns about the potential for drug resistance from taking PrEP medications.

“If you take things like… [PrEP] too early, you build up a tolerance or resistance and later on when you might really need it…” (age 62, White, HIV-positive, serodiscordant relationship).

Several participants offered specific options for the provision of PrEP by health care providers. Six individuals stated that PrEP should be limited to those at highest risk, such as young HIV-negative MSM or those who are not using condoms. The exclusive use of PrEP in serodiscordant couples was another option discussed by three participants.

“It should be offered to those who are sexually active at younger ages… but only if they're having sex with a lot of people” (age 49, Black, HIV-positive, seroconcordant relationship).

“I guess the ideal thing would be to find out what the person's personal habits are already… if someone is regularly using condoms and taking care to not get HIV, they're probably not the best candidate to sell this to because it may get them off the condoms; but if it is somebody that is going to bathhouses every weekend and they are HIV-negative, they should definitely have the option… my concern is that people that aren't at high risk may start to engage in more risky behavior” (age 44, White, HIV-positive, seroconcordant relationship).

“I'd be concerned [that] some people would think that it would mean that it would be okay to have any kind of risky… high risk behavior… on the other hand… if you are in a relationship where you're not in the same serostatus, it has a certain appeal because we both have a little bit of anxiety about when I get my every three-month HIV test… a little anxiety about ‘maybe… what if?’ and it would be nice if you could be less nervous about that.” (age 51, White, HIV-negative, serodiscordant relationship).

Although most participants agreed that PrEP should be offered to anyone who requests it, there were a few who were skeptical.

“I'm glad that they are coming up with ways to prevent HIV infection but there if a big need out there in the world for HIV meds for people who do have it [HIV] so I'm kind of skeptical about whether they should be giving it to people who don't have it yet.” (age 48, White, HIV-positive, seroconcordant relationship).

## Discussion

Our findings suggest that approximately 62% of participants in quantitative surveys and 81% of qualitative participants had heard of PrEP; however, qualitative data suggest that it is likely that these proportions are an overestimation and that about a quarter of these individuals confused PrEP with PEP. Therefore, reports of PrEP awareness among MSM may be overestimated and more effective messaging is necessary to educate individuals about the differences between PrEP and PEP.

In contrast to other studies [11], [23], approximately 40% of men and their partners in our quantitative study had low endorsement of PrEP uptake in the future and were ambivalent as to whether PrEP should be offered widely. Qualitative data indicate that this uncertainty was due to concerns about increased risk compensation, costs associated with PrEP medications, ARV adverse effects, the need for more public education and research, and potential for drug resistance. In contrast, roughly 48% of participants agreed or strongly agreed with the idea that PrEP should be offered to anyone who requests it. This high level of support may indicate the need for increased PrEP access, public education, and training of health care providers in appropriately prescribing PrEP, providing counseling and safer sex education, and monitoring for PrEP adverse effects or HIV seroconversion.

Behavioral disinhibition or risk compensation was the most frequently stated apprehension with the availability of PrEP. These concerns have been echoed in other publications [23], [24], [25], [26] and various models have suggested that small increases in risk behavior could potentially offset or reverse PrEP's benefits in reducing HIV infections at the population level [13], [14]. This concern is in contrast to the findings of the iPrEx study, which revealed a decrease in sex partners and an increase in condom use in both study arms [2]. However, in this trial, in addition to receiving frequent comprehensive counseling on the importance of condom use, participants were educated about the unproven efficacy of the active drug and the fact that they may be taking placebo. Therefore, it is difficult to discern whether risk compensation will arise with the introduction of PrEP and, as also voiced by our study participants, further research on the impact of PrEP on risk compensation and change in risk over time is crucial. In the absence of data on the real-world impact of PrEP on sexual risk behavior, frequent risk reduction counseling and discussion about sex practices is an essential part of PrEP delivery in a clinical setting.

It is notable that when asked about PrEP, participants focused primarily on troubling factors such as risk compensation, costs, and potential negative long-term health consequences, rather than possible positive aspects of PrEP, such as increased sexual enjoyment or intimacy that may accompany reduced condom use. Their cautionary words and occasionally moralistic statements may suggest that for men who cope with HIV on a daily basis, the potential renegotiation of sexual safety strategies trigger concerns and fears, perhaps due to a highly informed understanding of what HIV infection and its treatment entail. The tendency to express worry and doubt about PrEP may also be due to the participants' relatively older age, having lived through a havoc wreaked by AIDS in the 1980s and early 1990s. Their perception may have been that younger men who did not endure those years may hold cavalier views on PrEP.

A surprising finding among HIV-negative participants in our quantitative study was that those who reported UIAI with their HIV-positive primary partner were more likely to report possible future uptake of PrEP; however, the report of URAI with men other than primary partner who were HIV-positive/serostatus-unknown was negatively correlated with future PrEP use. Therefore, if we were to consider sexual risk behavior on a continuum, ranging from very low risk (no unprotected sex) to maximum risk (HIV-negative men having URAI with HIV-positive men); PrEP seemed to have been most appealing to HIV-negative MSM who were engaged in a moderate level of risk but not the highest risk. We hypothesize that this may have been due to the fact that those who were mindful of their risk may have already been engaging in low to moderate risk (i.e., seropositioning by UIAI with HIV-positive primary partner) and may have been more likely to want to further reduce their risk of HIV infection. Conversely, those at the highest risk (i.e., HIV-negative men reporting URAI with outside HIV-positive/unknown-serostatus partners) may have been less likely to be aware of their actual level of risk behavior or less concerned about becoming HIV infected. These findings point to one of the major dilemmas of PrEP that remains to be answered in future research: will the use of PrEP shift those who are already mindful of their risk and attempting to decrease their risk by strategies such as strategic positioning to a higher risk category?

One theory behind futile results of the FEM-PrEP trial is that the women who were included in this trial were at high risk of HIV infection but approximately 70% considered themselves to be at low risk or not at risk for HIV and thus this disconnect between behavior and risk perception may have influenced PrEP adherence [7]. Therefore, future research is needed to examine the efficacy of PrEP in MSM engaged in various levels of sexual risk, as well as their perception of HIV risk to determine the appropriate populations where PrEP can have the highest impact and groups in which more education and closer monitoring may be warranted. This is of paramount importance because any increase in risk behavior resulting from behavioral disinhibition among those who are more aware of their potential for HIV infection and currently engaged in low to moderate risk behavior could counteract or reverse any benefits from PrEP.

In our quantitative survey, younger age, lower education, and not being on ARVs were positively associated with HIV-positive men's beliefs that their partner would use PrEP and younger age was similarly related to HIV-positive men's level of agreement with offering PrEP to the community. HIV-positive participants on ARVs also raised concerns regarding PrEP toxicity and drug resistance in qualitative interviews. Cumulatively, these data indicate that those who were older, more educated, or on ARVs may have had an increased awareness of long-term ARV risks, had experienced adverse effects from taking older generations of ARV medications for longer durations, had a better grasp of adherence challenges and consequences of non-adherence in comparison to HIV-positive men who were younger, less educated, or not on ARVs. Although serious toxicities of tenofovir appear to occur infrequently and the majority of the events associated with TDF/FTC were mild or moderate in severity in the iPrEX study [2], long-term consequences of PrEP on bone and kidney health of HIV-negative individuals are unknown [27], [28]. Additionally, of the subjects who became HIV-positive during the iPrEX trial, no evidence of genotypic drug resistance was detected; however, very few had detectable drug levels at the time of seroconversion. Therefore, data from our study emphasize the importance of education of HIV-positive members of the community on the potential differences in the occurrence of adverse effects and drug resistance among HIV-negative individuals on PrEP versus those taking ARVs for HIV treatment in addition to the critical importance of adherence.

Two proposals by our qualitative study participants for the provision of PrEP by health care providers were to offer PrEP to those who are already engaged in high risk behavior and to those in serodiscordant relationships. The use of PrEP in HIV serodiscordant couples resulted in a 67-75% HIV risk reduction in the Partners PrEP study and is likely to be an important factor in the selection of appropriate candidates for PrEP use [3]. The prioritization of PrEP among MSM engaged in URAI has also been raised by other investigators [29]; however, the feasibility of identifying ideal “PrEP candidates” in a clinical setting and the efficacy of PrEP in these individuals, as well as the capacity of health care providers to monitor adverse effects and adherence [30] remains to be determined.

We used a mixed-methods approach to study knowledge, concerns, and correlates of future PrEP use in MSM living in San Francisco. This approach allowed us to complement our quantitative surveys with in-depth narratives from the qualitative interviews and to further explore participants' PrEP knowledge and concerns. A strength of this study is the utilization of couples-based data. Serodiscordant couples represent a potential target group for the implementation of PrEP and men in seroconcordant HIV-positive relationships may have outside partners or future primary partners who are HIV-negative. Therefore, understanding these individuals' level of awareness, attitudes, and concerns towards PrEP are important to assess and can contribute to better tailored messages and programs.

The design of the parent trial (i.e., the Duo Project) in which the qualitative interviews and quantitative survey were nested, limited our ability to utilize an iterative and integrated process between these two study arms. This parent trial also dictated the sampling for the two studies, in which the qualitative interviews had a slightly older population. Due to a certain level of confusion between PrEP and PEP, we do not know if our results would have been different had this confusion not existed. Ultimately, this confusion underscores the need for more education about HIV prevention approaches and the need for future research to clearly differentiate between these concepts. The goal of this study was primarily formative and exploratory; therefore, the uncovered themes and survey results may not be generalizable and representative of the entire MSM community in San Francisco or other cities worldwide.

Our findings have important implications for developing PrEP interventions as the effectiveness of PrEP will depend on the community's understanding of what PrEP is and their willingness to accept it as part of an HIV prevention strategy. Our data reveal the need for more intense efforts to introduce, educate, and gather feedback from MSM communities on the use, efficacy, cost, and toxicity of PrEP. This need also parallels the necessity to further train health care providers who will likely be the spokespersons for PrEP use in clinical settings. The appropriate use of PrEP in HIV-negative individuals and early initiation of ARVs in those living with HIV [31] have the potential to dramatically reduce HIV incidence. Our study findings suggest that there is much ambiguity about PrEP, ambivalence regarding future PrEP use, and apprehensions concerning the impact of PrEP on risk behavior. We believe that a comprehensive effort to educate, provide clinical care, and conduct clinical research is needed to overcome these issues.
